# Electrochemical Nanostructured Aptasensor for Direct Detection of Glycated Hemoglobin

**DOI:** 10.3390/ijms26157140

**Published:** 2025-07-24

**Authors:** Luminita Fritea, Cosmin-Mihai Cotrut, Iulian Antoniac, Simona Daniela Cavalu, Luciana Dobjanschi, Angela Antonescu, Liviu Moldovan, Maria Domuta, Florin Banica

**Affiliations:** 1 Faculty of Medicine and Pharmacy, University of Oradea, 10 P-ta 1 December Street, 410073 Oradea, Romania; lfritea@uoradea.ro (L.F.); daniela.cavalu@didactic.uoradea.ro (S.D.C.); dobjanschil@uoradea.ro (L.D.); aantonescu@uoradea.ro (A.A.); fbanica@uoradea.ro (F.B.); 2 Faculty of Material Science and Engineering, National University of Science and Technology Politehnica Bucharest, 313 Splaiul Independentei Street, District 6, 060042 Bucharest, Romania; cosmin.cotrut@upb.ro (C.-M.C.); iulian.antoniac@upb.ro (I.A.); 3 Faculty of Electrical Engineering and Information Technology, University of Oradea, 1 Universitatii Street, 410087 Oradea, Romania

**Keywords:** screen-printed carbon electrode, carbon nanotubes, gold nanoparticles, glycated hemoglobin, aptamer, electrochemical sensor

## Abstract

Glycated hemoglobin (HbA1c) is an important biomarker applied for the diagnosis, evaluation, and management of diabetes; therefore, its accurate determination is crucial. In this study, an innovative nanoplatform was developed, integrating carbon nanotubes (CNTs) with enhanced hydrophilicity achieved through cyclodextrin (CD) functionalization, and combined with gold nanoparticles (AuNPs) electrochemically deposited onto a screen-printed carbon electrode. The nanomaterials significantly improved the analytical performance of the sensor due to their increased surface area and high electrical conductivity. This nanoplatform was employed as a substrate for the covalent attachment of thiolated ferrocene-labeled HbA1c specific aptamer through Au-S binding. The electrochemical signal of ferrocene was covered by a stronger oxidation peak of Fe^2+^ from the HbA1c structure, leading to the elaboration of a nanostructured aptasensor capable of the direct detection of HbA1c. The electrochemical aptasensor presented a very wide linear range (0.688–11.5%), an acceptable limit of detection (0.098%), and good selectivity and stability, being successfully applied on real samples. This miniaturized, simple, easy-to-use, and fast-responding aptasensor, requiring only a small sample volume, can be considered as a promising candidate for the efficient on-site determination of HbA1c.

## 1. Introduction

Diabetes mellitus is a metabolic disease characterized by an increased blood glucose concentration due to a lack or insufficiency of insulin that regulates plasma glucose levels. It has become a worldwide epidemic disease with huge prevalence, attributed to onset factors such as unhealthy lifestyle and diet, as well as genetic causes. This non-transmissible metabolic disorder is usually implicated with complications such as retinopathy, nephropathy, neuropathy, and microvascular and cardiovascular disorders, resulting in morbidity and mortality for diabetic patients. This pathology has become a burden for the public health system, leading to an increased demand for diagnostic and monitoring tests, and indirectly to a significant demand for biosensors that detect disease onset and evolution with precision, speed, and comfort [1,2,3,4]. In order to avoid the complications of diabetes and to prolong the life expectancy of diabetics, it is very important to monitor blood glucose, which can be carried out by two methods: blood glucose determination (daily) and glycosylated hemoglobin determination (periodically) [1,2,3,4]. Non-glucose biomarkers such as glycated hemoglobin have become the target analyte for developing sensors that are useful for assessing diabetes type and predicting complications [1,2,3,4].

Glycosylated hemoglobin or glycated hemoglobin (HbA1c), a natural biologic product obtained after the spontaneous reaction of hemoglobin and glucose in blood, is an important biomarker used for the diagnosis and management of diabetes, showing the average blood glucose level for the previous two–three months. The HbA1c level (usually expressed in percentage, %) is referred to as a ratio between the glycated hemoglobin and the total hemoglobin, with normal values in the range of 4–6%, while levels higher than 6.5% indicate diabetes. According to the American Diabetes Association, the recommended values of HbA1c indicating good glycemic control during therapy should be less than 7% for adult diabetics.

Among the traditional analytical methods currently employed for HbA1c determination, chromatographic, electrophoretic, enzymatic and immunological methods have been widely and successfully used in the last decade, but several drawbacks emerged: high cost, time consuming, and advanced technical skills required for handling [3,4]. Although nowadays ion-exchange HPLC is considered the gold standard for HbA1c determination, electrochemical methods have recently emerged, showing multiple advantages over other techniques, such as sensitivity, low cost, simplicity, real-time results, and miniaturization. Most sensors are based on boronic acid, antibodies, and enzymes [5,6,7]. Lately, specific aptamers for HbA1c and nanomaterials have been used for the biofabrication of various aptasensors [8,9,10,11,12,13,14,15,16,17,18], showing excellent sensitivity and selectivity. Various aptasensors reported for HbA1c determination have employed carbon-based nanomaterials, metallic nanoparticles, and metal–organic frameworks as electrode modifiers and used voltammetry and amperometry as electrochemical methods of detection, leading to improved analytical performances [8,9,10,15,16,17,18].

Direct-type electrochemical sensors for HbA1c determination involve selective binding of the protein target through the bio-affinity of boronic acid, ferrocene, aptamers, and antibodies, leading to changes in current, voltammetry, potential, and impedance; therefore, these sensors are classified as amperometric sensors, voltametric sensors, potentiometric sensors, and impedimetric sensors [19,20,21]. The future trend should be focused on the elaboration of point-of-care (PoC) tests for HbA1c detection by small, portable, and commercialized analytical tools, which should become available in clinical practice as standard glucose tests, serving as ‘anywhere-anytime-anyone’ devices [14,21].

Aptamers are single-stranded oligonucleotides synthesized by the SELEX procedure (systematic evolution of ligands by exponential enrichment) that have proven their superiority over antibodies due to their wide range of unique and advantageous properties: stability, improved cost, industrial chemical synthesis, ease of modification, capacity to recognize virtually any class of target molecules with high affinity and specificity, and their reversible configuration [22,23]. Aptamers can be site-specifically modified in order to incorporate linkers or other functional groups on both the phosphate/ribose backbone and the nucleobases, in order to improve resistance to enzymatic degradation, reduce off-target events, and become immobilized on substrates for various applications [22,23]. Recent progress was recorded in the application of these functional nucleic acids as novel sensors for biomolecular detection, drug discovery, drug delivery, and bio-nanotechnology [24,25,26,27,28,29].

Combining nanotechnology with biosensing is crucial for PoC diagnosis; hence, carbon-based nanomaterials such as fullerenes (0D), CNTs (1D), and graphene (2D) are broadly employed in healthcare biosensing [30,31,32], with the advantage of localized detection along with real-time and label-free non-destructive sensing.

CNTs (either single-walled carbon nanotubes—SWCNTs, or multi-walled carbon nanotubes—MWCNTs) possess unique structural, electronic, and chemical properties: high surface area, high electrical and thermal conductivity, excellent electrocatalytic properties, and excellent mechanical resistance [30,31,32]. On the other hand, nanoparticles such as AuNPs, silver nanoparticles (AgNPs), and platinum nanoparticles (PtNPs), which possess a large surface area and exhibit unique optical properties, are also materials of choice for electrochemical biosensor design due to their advantageous properties such as the immobilization of biomolecules while preserving their biological activity and creation of efficient conducting interfaces with electrocatalytic ability [30,31,32].

Cyclodextrins (CDs), non-reducing cyclic oligosaccharides, have a relatively hydrophobic internal cavity, forming inclusion compounds between CDs and different molecules (through host-guest interactions), and a hydrophilic exterior, leading to improved dispersibility and molecular recognition [33,34]. The combination of nanomaterials and CDs has been approached in many studies for developing various sensors with improved performances [33,34].

Screen-printed electrodes (SPEs) based on various materials (carbon, gold, platinum, etc.) are widely used in (bio)sensor design due to advantages such as their miniaturized form, which allows the use of reduced volumes of solutions (μL); their single use electrodes, minimizing blood contamination; and their small size and portability, making them suitable for PoC devices [32,35]. The basic fabrication principles and configuration design of SPEs for electrochemical applications allowed the determination and identification of drugs, pathogenic microorganisms, viruses, and biomarkers for clinical analysis purposes [32,35]. The working electrodes are made of different conductive materials, with multiple possibilities for their modification using various nanomaterials such as graphene, carbon nanotubes, metal nanoparticles, or quantum dots [32,35].

A key point in the elaboration of biosensors is considered to be the interactions between the biological elements and their targets; hence, in the case of aptasensors, the immobilization of aptamers represents a real challenge. From a technical point of view, several immobilization methods at the electrode surface are currently used: direct attachment, covalent binding to surface, biocoating, and hybridization using complementary oligonucleotides [35].

A further increase in diabetic persons is expected worldwide; therefore, the accurate and accessible detection of HbA1c is crucial for the diagnosis and management of this metabolic disease. In this context, the aim of our work was the elaboration of a novel electrochemical aptasensor based on nanomaterials (CNTs and AuNPs) for the sensitive and selective determination of HbA1c using a simple, low-cost, and accurate method that is suitable for PoC analysis. The HbA1c aptamer was covalently functionalized with a thiol group (-SH) at the 5′-end for attachment through Au-S binding, and with Ferrocene (Fc) at the 3′-end for redox signal. Herein, we elaborate an innovative platform consisting of CNTs functionalized with CD for a better hydro dispersion, combined with electrodeposited AuNPs; these nanomaterials were adopted in order to enhance the electrochemical performance of the SPE. The nanoplatform characterization was performed by electrochemical and microscopic methods. Then, a thiolated ferrocene-labeled aptamer for glycated hemoglobin was immobilized through linkage to the gold nanostructures. Eventually, the ferrocene-labeled aptamer specifically bound to HbA1c molecules, leading to the modification of the Fc electrochemical signal, which was covered by a stronger oxidation signal of Fe^2+^ from HbA1c structure (HbA1c contains four atoms of Fe^2+^, which are electroactive). Therefore, it achieved direct electrochemical detection of the protein target. The proposed nanostructured aptasensor demonstrated good analytical performance, such as low cost, simplicity of fabrication, fast response, sensitivity, selectivity, reproducibility, stability, recovery, a wide linear range, and an acceptable limit of detection, presenting potential applicability in diabetes management and screening.

## 2. Results and Discussion

### 2.1. Electrochemical Characterization of the Nanoplatform

**Scheme 1:**
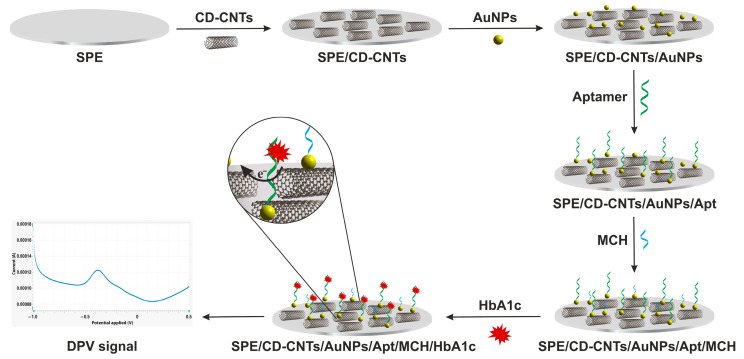
Graphical representation of the aptasensor elaboration process: drop coating of SPE with CD-CNTs; electrochemical generation of AuNPs; aptamer immobilization; blocking step with MCH; and HbA1c immobilization.

The elaboration process of the aptasensor is presented in Scheme 1. First, the SPE was modified with CD-CNTs by drop coating, then it was covered by electrochemically deposited AuNPs. On this nanostructured platform, the thiolated ferrocene-labeled aptamer was immobilized via Au-S bounds, and MCH was then used in order to block non-specific adsorption. Lastly, the HbA1c target was immobilized and the interaction between the ferrocene-labeled aptamer and HbA1c generated changes in the electrochemical signal of Fc, which was eventually covered by a stronger Fe^2+^ signal from HbA1c structure.

**Figure 1:**
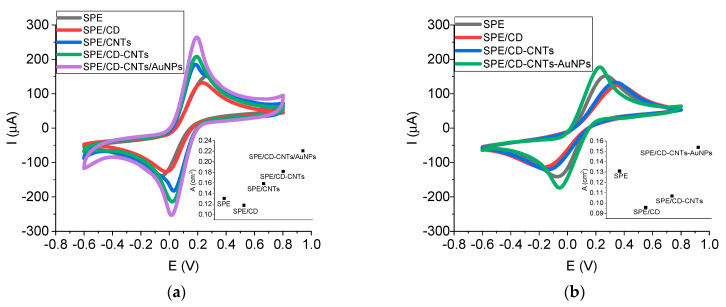
(**a**) CVs in 5 mM [Fe(CN)_6_]^3−/4−^ on different platforms (drop coating) (100 mV/s); (**b**) CVs in 5 mM [Fe(CN)_6_]^3−/4−^ on different platforms (electropolimerization) (100 mV/s). Insets: variation in the active surface area of the different platforms.

SPEs were in-lab functionalized with various modifiers in order to increase the analytical performance. Two techniques were employed: the drop method (Figure 1a) and electropolimerization method (Figure 1b). Cyclic voltammograms (CVs) were performed after each SPE modification and the active surface area was also calculated in order to indicate the influence of each added modifier for both techniques (Figure 1a,b insets).

When the drop coating method was used, the intensity of the peak current increased with adding CNTs (in comparison with bare SPE) due to the high surface area and excellent conductivity of the CNTs. Moreover, the increase was even higher (with around 40%) when CD was also added in order to enhance the CNTs’ dispersibility in aqueous medium. SPE functionalization with only CD (in considerably high concentration) did not improve the electrode conductivity (the low conductivity of CD led to passivation). The purpose of using CD was to improve the CNTs’ dispersion in water, leading to a more concentrated suspension. The electrodeposition of AuNPs increased the electrode conductivity even more (with around 70% in comparison with bare SPE); therefore, the best configuration (SPE/CD-CNTs/AuNPs) contained both nanomaterials that increased the electrode surface area and the electrical conductivity.

The variation in peak-to-peak separations (ΔE) followed the same trend: from 0.323 V for bare SPE, it decreased to 0.174 V in the case of SPE/CD-CNTs/AuNPs, highlighting that the nanomaterials improved the electron transfer kinetics. Therefore, the nanoplatform SPE/CD-CNTs/AuNPs exhibited a superior electrocatalytic performance.

**Figure 2:**
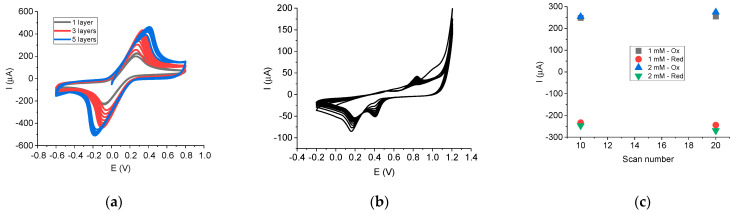
(**a**) CVs in 5 mM [Fe(CN)_6_]^3−/4−^ on SPE modified with 1, 3, 5 layers of CD-CNTs (drop coating) (100 mV/s); (**b**) CV of AuNP electrochemical generation on SPE/CD-CNTs (1 mM HAuCl_4_, 10 scans); (**c**) variation in peak intensity with different parameters for AuNP electrodeposition (1 mM, 2 mM, 10 scans, 20 scans) (CV in 5 mM [Fe(CN)_6_]^3−/4−^).

The electrochemical generation of AuNPs was achieved by using CV, and increasing peaks at 0.84 V (when scanning the potential towards oxidation) and at 0.40 V and 0.15 V (when scanning the potential towards reduction) were noticed (Figure 2b).

When the electropolimerization method was employed, a slow increase (of around 20% in comparison with bare SPE) was observed only in the case adding all three modifiers (CD-CNTs-AuNPs); meanwhile, the ΔE decreased to 0.277 V. Therefore, the modification of SPE in one step by the electropolymerization of all modifiers proved not as efficient as the previous method, maybe due to a steric hindrance of nanomaterial deposition/generation on the SPE, leading to a lower surface area with lower exposure to the redox probe.

According to these results, the nanoplatform SPE/CD-CNTs/AuNPs obtained by drop coating were chosen for further elaboration of the aptasensor.

Other optimized parameters included the number of CD-CNTs layers (Figure 2a) and electrodeposition of AuNPs (concentration and scans number) (Figure 2c). The deposition of different layer numbers of CD-CNTs was investigated (Figure 2a). The peak intensity increased by around 40% (3 layers) and by around 70% (5 layers) in comparison with 1 layer (evaluating in all cases the third scan from CVs). However, by increasing the number of layers, the penetration of the redox species and electrolyte was slowed down: only at around 5–8 scans was the signal almost stabilized for 3–5 layers, in comparison with 2–3 scans for 1 layer. After stabilization, the peak intensities obtained for 3 and 5 layers were almost the same. These results can be explained: by increasing the CD-CNTs layers, dense deposits of CD-CNTs are formed, thus increasing the distance to the electrode surface and leading to constraints towards the diffusion [36].

The ΔE also significantly increased to 0.434 V for 3 layers and to 0.583 V for 5 layers at signal stabilization, suggesting that electron transfer resistance was augmented by the increase in CD-CNTs layers, possible due to the high amount of CD affecting the conductivity [37].

Therefore, increasing the number of layers is not justified considering the hindered penetration of the electrolyte and slowed electron transfer rate. Moreover, the choice of 1 layer is less time-consuming and less resource-consuming to apply. Consequently, the nanoplatform obtained after dropping 1 layer of CD-CNTs was chosen for further optimization.

Concerning the optimization of the electrochemical generation of AuNPs, various parameters were tested: 1 mM and 2 mM concentrations of HAuCl_4_, with 10 scans and 20 scans for each concentration (Figure 2c). In the case of electrodeposition using 1 mM HAuCl_4_ and 20 scans, a slight increase in the anodic peak intensity was recorded (with around 7 µA) in comparison with 10 scans. In the case of 2 mM HAuCl_4_, the increase was around 5 µA (for 10 scans) and 26 µA (for 20 scans, meaning an increase of 10%) in comparison with 1 mM HAuCl_4_, at 10 scans. Therefore, the intensity improvement was not significant when increasing HAuCl_4_ concentration and scan number for electrodeposition, so the final configuration consisted of 1 mM HAuCl_4_ and 10 scans.

**Figure 3:**
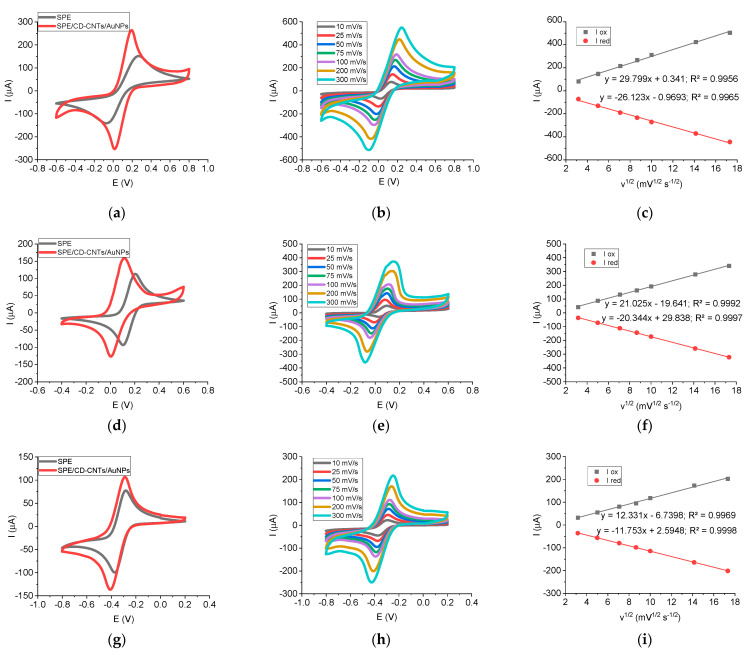
(**a**,**d**,**g**) CVs on SPE, SPE/CD-CNTs/AuNPs (100 mV/s), (**b**,**e**,**h**) CVs at various scan rate on SPE/CD-CNTs/AuNPs, variation in peak intensity vs. the square root of the scan rate from (**b**,**e**,**h**) for (**a**–**c**) 5 mM [Fe(CN)_6_]^3−/4−^, (**d**–**f**) 5 mM Fc(MeOH)_2_, and (**g**–**i**) 5 mM Ru(NH_3_)_6_Cl_3_.

The electrochemical behavior of SPE/CD-CNTs/AuNPs was evaluated in comparison with bare SPE in the presence of three redox probes: [Fe(CN)_6_]^3−/4−^, Fc(MeOH)_2_, and Ru(NH_3_)_6_Cl_3_ (Figure 3). The tests with the three different redox probes aimed to determine the surface characterization in terms of charge loading [38]. The ferro/ferric probe was negative, the ferrocene probe was neutral, and the ruthenium probe was positive. In all cases, higher cathodic and anodic peak currents were recorded on the nanoplatform compared with the bare SPE. This can be ascribed to the higher surface area and improved electrical conductivity of the nanomaterials used for SPE modification. The highest intensity increase was recorded for the negatively charged [Fe(CN)_6_]^3−/4−^ probe (with around 100 µA), and the lowest one for the positively charged Ru(NH_3_)_6_Cl_3_ probe (with around 20 µA). A higher signal for the negative probe would indicate a positive surface charge, which attracts negative species in solution, while a lower signal for the positive probe would suggest a positive surface charge and thus repulsion for those compounds in solution.

Concerning the potential, the biggest ΔE variation between the two types of SPEs was recorded for [Fe(CN)_6_]^3−/4−^ probe, with ΔE being lower in the case of the nanoplatform (indicating an enhanced electron transfer rate). On the other hand, for the other two redox probes, the ΔE variation was very low, being a little bit higher in the case of the nanoplatform.

Therefore, the best electrochemical response of the SPE/CD-CNTs/AuNPs was registered for the negatively charged [Fe(CN)_6_]^3−/4−^ probe.

In order to establish if the process at the electrode surface is governed by adsorption or diffusion of the redox species, CV scans were recorded at different scan rates. A good linear correlation between peak current intensity and the square root of the scan rate indicates a diffusion-controlled process with the analyte freely diffusing in solution towards the electrode [39,40]. The current intensities of the electrochemical oxidation and reduction signals of the three redox probes were plotted vs. the radical of scan rate. The anodic and cathodic peak intensity varied linearly with the square root of the scan rate, with good correlation coefficients for all three redox probes (Figure 3c,f,i), indicating that the electrochemical processes were controlled by the diffusion of the analyte towards the electrode surface.

### 2.2. Microscopic Characterization of the Nanoplatform

**Figure 4:**
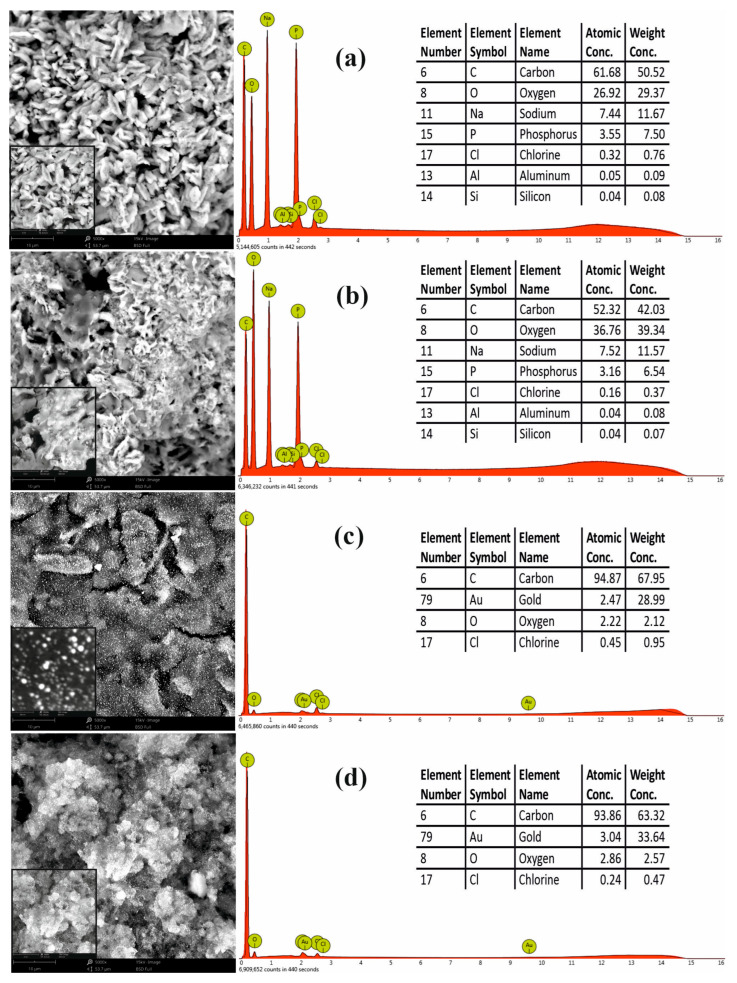
SEM images and EDS spectrum with the corresponding tables for (**a**) SPE/CNTs (scale bar 10 μm, inset scale bar 8 μm), (**b**) SPE/CD-CNTs, (scale bar 10 μm, inset scale bar 8 μm); (**c**) SPE/AuNPs, (scale bar 10 μm, inset scale bar 500 nm); and (**d**) SPE/CD-CNTs/AuNPs (scale bar 10 μm, inset scale bar 8 μm).

In order to confirm the modification of SPEs, the nanoplatforms were also characterized using SEM and EDS methods. The surface morphology and element composition of the nanomaterials deposited on SPEs are presented in the SEM images and EDS spectrum below (Figure 4). According to the manufacturer, the MWCNTs’ average length was 1.5 µm. Figure 4a,b reveal the typical structural details of CNTs deposited on the SPE prior to and after mixing with CD (the high concentration of CD leads to a crowded CNTs network).

Figure 4c presents the morphological details (inset—details recorded with high magnification 135,000×) of AuNPs electrodeposited on SPE, showing a spherical shape and diameter size under 100 nm (in accordance with our previous work where we employed the same electrochemical protocol deposition [41]). The nanostructures resulting from the electrodeposition of AuNPs on CD-CNTs are presented in Figure 4d. A homogenous and uniform distribution of spherical AuNPs on the surface of CD-CNTs scaffold can be observed, providing a large surface area for aptamer immobilization and also for facilitated electron transfer.

The EDS spectrum indicated, apart from carbon, oxygen, and gold (related to CNTs, CD, and AuNPs), traces of Na^+^, Cl^−^, and P resulting from the used buffers (PBS, acetate buffer).

### 2.3. Elaboration and Characterization of the Aptasensor

**Figure 5:**
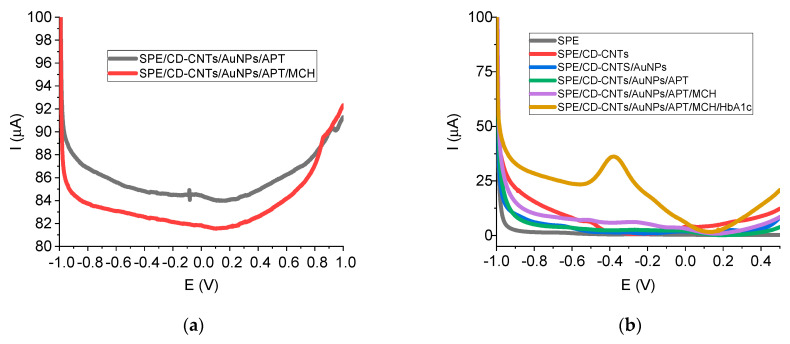
(**a**) DPVs in TRIS buffer (0.1 M TRIS, pH 7.5) on the nanoplatform modified with aptamer (in the absence and presence of MCH) and (**b**) on bare SPE and on different types of modified SPEs.

All the steps included in the protocol for aptasensor elaboration were characterized and optimized by using DPV in TRIS buffer (Figure 5 and Table 1). DPV was recorded after each step of SPE modification. Thiolated aptamer used as a bioreceptor for HbA1c was immobilized at the electrode surface by strong covalent bonds with AuNPs.

The oxidation peak of Fc from aptamers labeled with this redox probe is usually situated at positive values of potential (around +0.1 V, +0.2 V, or +0.35 V, respectively) [15,42,43,44]. In our case, the Fc peak was very close to 0 V (at low values below 0 V), with a low intensity (under 1 µA) (Figure 5a), decreasing after MCH blocking (Figure 5a). As can be observed in Figure 5a, the redox signal for gold from AuNPs deposition exists at around 0.9 V.

As can be observed in Figure 5b, only after the incubation of the protein was a huge oxidation peak obtained (around 20 µA at around −0.37 V for 5.5% HbA1c) due to the affinity capture of the target by the aptamer, indicating a direct electrochemical detection of HbA1c. This redox signal was attributed to the electroactive couple Fe^2+^/Fe^3+^ from heme proteins (such as hemoglobin and glycated hemoglobin). Other studies have also studied and reported the direct electron transfer process of hemoglobin on the electrode surface at around −0.3 V ~ −0.5 V, and some of these sensors employed Nafion and chitosan membrane for hemoglobin or red blood cell immobilization [45,46,47,48,49]; meanwhile, others have reported the use of electron transfer mediators [9].

The small Fc oxidation peak was covered by the higher oxidation peak of Fe^2+^ from the protein target, HbA1c. Therefore, the well-defined peak of the direct electrochemical oxidation of Fe^2+^ from the HbA1c structure recorded at around −0.37 V was used in subsequent electrochemical experiments.

The optimization protocol for the aptasensor elaboration included various analytical parameters (concentration, time, temperature) for aptamer immobilization, MCH blocking, and HbA1c immobilization, as presented in Table 1. Concerning the aptamer immobilization, the increase in aptamer concentration did not significantly increase the signal, as 1 µM and 5 µM aptamer generated similar intensities, which may indicate a saturation of the electrode surface with the aptamer [16]. Therefore, the concentration of 1 µM aptamer was chosen. A higher concentration of aptamer (10 µM) resulted in a slowly reduced signal, maybe due to an unfavorable immobilization of the aptamer or an unfavorable removal of captured HbA1c by washing, a behavior also reported by Jaberi et al. [10].

The aptamer immobilization at 25 °C generated the lowest signal; meanwhile, that at 4 °C was determined to be the highest intensity, being a little bit higher compared to the one at 37 °C. Thus, the temperature of 4 °C was selected as the optimal condition (the choice was also made considering that the immobilization at refrigerator temperature is more effective as evaporation is avoided). Time was also optimized for aptamer immobilization: the difference between 60 min and 30 min was not significant; therefore, 30 min was used for further analysis.

The next step assessed was blocking with MCH in order to reduce non-specific adsorption on the electrode surface. By increasing the MCH blocking time (15 min to 30 min), the signal very slowly decreased; thus, 15 min was chosen as it was more time-effective.

The optimization of the protein target immobilization evaluated the influence of temperature and of time. Incubation at 4 °C generated the lowest current intensity; meanwhile, between 25 °C and 37 °C, there was no significant difference. Taking into account that incubation at 37 °C is more resource-consuming (it is mandatory to use an oven), the room temperature of 25 °C was selected for HbA1c incubation, which is also more suitable for PoC devices. Concerning time as a parameter, the same very slight difference in intensity was recorded between 60 min and 30 min for protein incubation; therefore, the 30 min incubation was considered to be optimum.

**Figure 6:**
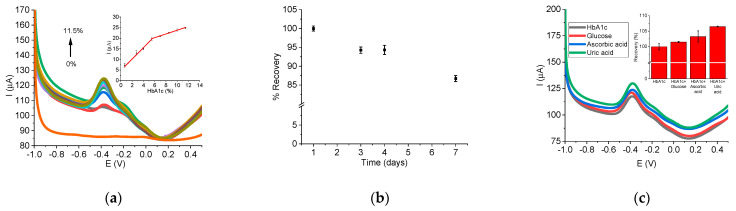
(**a**) DPVs for increasing the concentration of HbA1c on the aptasensor (0, 0.688, 1.375, 2.75, 4, 5.5, 7, 8.5, 10 and 11.5%); inset: variation in the peak intensity with HbA1c concentration. (**b**) The stability over time and (**c**) interference studies of the aptasensor.

To investigate the relationship between the electrochemical signal and the protein target concentration, the aptasensor response to various concentrations of HbA1c was recorded by using the two HbA1c controls (also performing dilutions). The current signal of HbA1c presented two linear ranges, from 0.688% to 5.5% and from 5.5% to 11.5% (Figure 6a). The linear dependence yielded two regression equations: I (µA) = 5.288 + 2.593 [C] [%] (R^2^ = 0.9903) and I (µA) = 14.881 + 0.889 [C] [%] (R^2^ = 0.9981). Thus, the corresponding sensitivities were 2.593 µA/% and 0.889 µA/%, respectively. The linear range was limited to this value (11.5%) because that was the highest concentration of the control solutions. Therefore, the two concentration ranges on which the aptasensor can be applied are suitable both for diabetic and non-diabetic persons. The limit of detection (LOD) was calculated to be 0.098% (calculated as three times the standard deviation of the blank divided by the slope of the calibration curve), and the limit of quantification (LOQ) was 0.325%.

The assessment of other characteristics of the proposed aptasensor were investigated, leading to excellent reproducibility (RSD 3.94%, where *n* = 3 for 5.5%, *n* = 3 for 11.5%) and good stability over a week (around 90% of the signal was maintained) (Figure 6b). The aptasensor also presented very good selectivity in the presence of some common interfering species, with 100 µM concentration (101.49% ± 0.05 recovery in the case of glucose, 103.26% ± 1.80 for ascorbic acid, 106.48% ± 0.16 for uric acid) (Figure 6c) and significantly good recovery from real samples (from 93.09% to 98.44%) (Table 2). Taking into account that the aptasensor for HbA1c determination is envisaged to be used in real blood samples, the solution to avoiding contamination was the use of a single-use disposable platform. Therefore, considering the disposable nature of the SPE-based sensor, the repeatability using a single sensor is not necessary and has not been evaluated.

All these results highlight that the aptasensor based on nanomaterials (CD-CNTs, AuNPs) can be successfully employed for selective HbA1c determination as an alternative method with clinical application for patients with diabetes. Taking into account the small size, low sample volume, and wide linear detection range, if this SPE aptasensor was integrated into a portable electrochemical device, it could generate a PoC tool for diabetes monitoring for home users.

Compared with some other electrochemical aptasensors reported in the literature for HbA1c determination (Table 3), the proposed nanostructured aptasensor presents low cost and simplicity of fabrication. The steps involved to create this aptasensor were fewer and more simple in comparison with others, which employed poly (2,2′:5′,5″-terthiophene-3′-p-benzoic acid) and EDC/NHS coupling [9], or Cobalt(II) metal–organic framework/two-dimensional molybdenum diselenide in a sandwich-like immunosensor configuration [18]. Moreover, this electrochemical aptasensor was superior to others concerning the direct detection based on the electrochemical signal of the target protein; meanwhile, other studies used the redox signal of [Fe(CN)_6_]^3−/4−^ [8,10,16], Fc [15], electrocatalytic reduction current of toluidine blue O [9], Ag oxidation [17], or SiW_12_ redox indicator [18]. To the best of our knowledge, this nanoplatform based on two types of nanomaterials (CNTs and AuNPs) represents the first reported electrochemical nanostructured aptasensor achieving the direct detection of HbA1c. Other advantages can be mentioned: fast response (the incubation time with aptamer, MCH, and target is lower than in other studies), wide linear range, acceptable LOD, quite good sensitivity, and excellent recovery. These features qualify it for clinical applications in the biomedical field for the better on-site care of diabetes patients.

## 3. Materials and Methods

### 3.1. Reagents and Materials

All reagents were of analytical grade and were used as received. Multi-walled carbon nanotubes were purchased from Nanocyl (Ref.: Nanocyl 3100, Sambreville, Belgium), β-cyclodextrin from Calbiochem (San Diego, CA, USA), and HAuCl_4_ from Emsure (Merk, KGaA, Darmstadt, Germany). All the other reagents (Na_2_HPO_4_, NaH_2_PO_4_, CH_3_COOH, CH_3_COONa, tris(hydroxymethyl)aminomethane (TRIS), NaCl, KCl, MgCl_2_; K_3_[Fe(CN)_6_], K_4_[Fe(CN)_6_], Fc(MeOH)_2_, Ru(NH_3_)_6_Cl_3_, mercaptohexanol (MCH), glucose, ascorbic acid, and uric acid) were purchased from Sigma-Aldrich (Merck KGaA, Darmstadt, Germany). Phosphate-buffer solution (PBS, pH 7.4, 0.1 M) was used for the preparation of [Fe(CN)_6_]^3−/4−^, Fc(MeOH)_2_, and Ru(NH_3_)_6_Cl_3_ solutions (5 mM of each). HAuCl_4_ solution (1 mM, 2 mM) was prepared by using acetate buffer (pH 4.5, 0.1 M). All aqueous solutions were prepared with ultrapure water (Fistreem Cyclon, Cambridge, UK). β-CD solution (15 mg in 1.5 mL PBS), CNTs suspension (1.5 mg in 1.5 mL PBS), CD-CNTs suspension (15 mg β-CD, 1.5 mg CNTs in 1.5 mL PBS), and CD-CNTs-HAuCl_4_ suspension (15 mg β-CD, 1.5 mg CNTs in 1.5 mL HAuCl_4_ 1 mM) were dispersed by ultrasonication for 90 min. The ratio of 1:10 between CNTs and β-CD was previously used in other studies [50,51,52].

HbA1c DNA aptamer (5′-TGGCAGGAAGACAAACACATCGTCGCGGCCTTAGGAGGGGCGGACGGGGGGGGGCGTGGTCTGTGGTGCTGT-3′, 5′-end thiol-Spacer C6 modification, 3′-end ferrocene modification, HPLC purification, 200 nmol scale) was synthesized by Alpha DNA (Montreal, QC, Canada). The aptamer sequence (72 bases) was selected from the literature based on its affinity for the target analyte [6]. Thus, the reported value for the HbA1c aptamer affinity was 7.3 nM (Kd), and some other non-target proteins (Hb, BSA, myoglobin) showed no significant interferences [6]. The aptamer specificity for HbA1c (the discrimination from other Hb molecules) was achieved by the method employed for aptamer selection, the on-chip SELEX process using magnetic beads (Hb-coated beads as a negative selection step, and HbA1c-coated beads as a positive selection step) [6,53]. Competitive and equilibrium binding assays were employed for the examination of the HbA1c aptamer, highlighting its binding affinity and specificity [53]. Some factors are crucial for the high binding affinity and specificity of aptamers, such as their specific 3D shape and binding with the target protein through van der Waals interactions and hydrogen bonds [53].

The HbA1c aptamer was dissolved in 83 µL of TRIS buffer (0.1 M TRIS, 0.1 M NaCl, 0.1 M KCl, 0.005 M MgCl_2_, pH 7.5), resulting in 100 µM stock solution; dilutions were performed by using the same Tris buffer solution. All the aptamer aliquots were stored at −20 °C. Before each experiment, the aptamer solution was thermally denatured (5 min at 90 °C, 2 min at −20 °C) according to Tertis M. et al. [42]. Glycosylated hemoglobin (HbA1c, control 1 = 5.5%, control 2 = 11.5%) was purchased from Trinity Biotech (Kansas City, MO, USA) and dilutions were performed with Trinity Biotech Diluent and kept refrigerated. Human serum was purchased from Beckman Coulter (Brea, CA, USA) and prepared by adding 5 mL of fresh deionized water; then, a dilution of 1:100 was used for real samples analysis; the solution was kept refrigerated.

### 3.2. Electrochemical Measurements

The electrochemical measurements were carried out on a potentiostat-galvanostat PG-STAT 128N with Nova 2.1.6 software (Metrohm Autolab, Utrecht, The Netherlands), using screen-printed carbon electrodes (110, Dropsens, Metrohm). The screen-printed carbon electrodes consisted of a three-electrodes system: carbon as the working electrode (WE, diameter 0.40 cm), carbon as the auxiliary electrode, and silver as the reference electrode. The electrochemical methods employed were cyclic voltammetry (CV) and differential pulse voltammetry (DPV), at room temperature. The electrochemical measurements were performed in duplicate and the error bars represent the standard deviation for those tests.

The SPEs were modified with various configurations: β-CD solution, CNTs suspension, CD-CNTs suspension, and CD-CNTs-HAuCl_4_ suspension. Two methods were investigated: drop coating and electropolimerization in one step. For drop coating, 10 µL was dropped on the WE and then dried before analysis. The electropolimerization method employed the following parameters: 40 µL dropped on the three-electrodes system, cycling the potential between −1 V and +1 V, with a scan rate of 100 mV/s for 10 scans; and between −1 V and +1.2 V, with a scan rate of 100 mV/s for 10 scans for AuNP electrodeposition, respectively. The elaborated platforms were then tested in [Fe(CN)_6_]^3−/4−^ by CV (−0.6 V, +0.8 V, 100 mV/s, 3 scans) for comparison. The optimized nanoplatform (SPE/CD-CNTs/AuNPs) was analyzed in the presence of three redox systems ([Fe(CN)_6_]^3−/4−^, Fc(MeOH)_2_, and Ru(NH_3_)_6_Cl_3_) by using CV with different scan rates (10–300 mV/s). The electrochemical deposition of AuNPs on the SPE/CD-CNTs was achieved by CV, according to our previous work (−0.2 V, +1.2 V, 100 mV/s, 10 scans) [41], optimizing the concentration of HAuCl_4_ (1 mM and 2 mM) and the number of scans (10 scans and 20 scans).

The aptasensor elaboration consisted of more successive steps. The nanoplatform was designed as follows: 10 µL of CD-CNTs suspension was dropped on WE and dried; then, 40 µL of 1 mM HAuCl_4_ was dropped on SPE for AuNP electrodeposition. Next, 5 µL of aptamer was dropped on WE (various concentrations of 1 µM, 5 µM, 10 µM) and incubated for a certain period of time (30 min, 60 min) at a certain temperature (4 °C, 25 °C, 37 °C), avoiding evaporation. Afterwards, 5 µL of MCH was dropped on WE (1 mM, 4 °C) for a certain period of time (15 min, 30 min). Finally, 5 µL of the protein target was dropped on WE (different concentrations) and incubated for a certain period of time (30 min, 60 min) at a certain temperature (4 °C, 25 °C, 37 °C), avoiding evaporation (kept in a high-humidity atmosphere). After each step, the SPE was thoroughly rinsed with buffer solution in order to eliminate the unbound molecules.

The tests were performed in TRIS buffer by using DPV with the following parameters: start potential −1 V, stop potential 0.5 V, step 0.001 V, modulation amplitude 0.05 V, modulation time 0.05 s, interval time 0.1 s. For the stability assessment, the aptasensors were elaborated in the same day, kept at 4 °C, and then tested in protein solution (11.5%) on different days. The selectivity tests were performed in the presence of 0.1 mM interference substances (glucose, ascorbic acid, uric acid). For the real sample analysis, the human serum was spiked with a known concentration of protein and the recovery rates were calculated.

### 3.3. Microscopic Measurements

The morphological characteristics and chemical composition of the aptasensor were analyzed with a scanning electron microscope equipped with an X-ray energy-dispersive spectrometer (SEM-EDS—Phenom ProX, Phenom World, Eindhoven, The Netherlands). The images were acquired using a backscattered electron detector (BSED). Due to the non-conductive nature of the aptasensor, a specialized charge-reduction holder was utilized to obviate the necessity for additional sample preparation of non-conductive samples.

## 4. Conclusions

A novel platform based on SPE modified with drop-coated CD functionalized CNTs and electrogenerated AuNPs was developed by a simple protocol and characterized by electrochemical and microscopic methods. Afterwards, it was employed as a basis for the elaboration of an electrochemical aptasensor for the direct determination of glycated hemoglobin, with good analytical performance (wide linear range, acceptable limit of detection, and good sensitivity, selectivity, reproducibility, stability, and recovery). By combining the specific features of nanomaterials, aptamers, and electrochemical methods, the elaborated aptasensor presented many advantages, such as a simple, miniaturized, easy-to-use, low-cost, fast-response design, small sample volume requirement, and sensitivity and selectivity. Therefore, the proposed nanostructured aptasensor is suitable for PoC analysis for diabetic persons. However, apart from the promising results obtained, the following features should be addressed in prospective studies: longer intervals for stability tests, comparison with laboratory standard method, and applicability in diagnostic practice. Future research will focus on extending the range of detectable biomarkers in order to elaborate a multi-target sensor.

## Figures and Tables

**Table 1:** DPV parameters obtained during aptasensor optimization (the parameters in bold are those chosen for further experiments).

Elaboration Step		Optimized Parameters		I/μA
Aptamer immobilization	MCH 1 mM, 4 °C, 30 min HbA1c 60 min, **25 °C**	Aptamer concentration (60 min, 25 °C)	**1 μM**	19.92 ± 0.31
			5 μM	21.19 ± 0.74
			10 μM	18.44 ± 0.45
		Temperature	**4 °C**	23.84 ± 1.25
		(1 μM, 60 min)	37 °C	23.25 ± 1.13
		Time (1 μM, 4 °C)	**30 min**	23.61 ± 0.96
MCH blocking	HbA1c 60 min, 25 °C	Time (**1 mM, 4 °C**)	**15 min**	24.26 ± 0.47
HbA1c immobilization		Temperature	4 °C	20.39 ± 1.04
			37 °C	23.69 ± 0.27
		Time	**30 min**	24.49 ± 1.23

**Table 2:** Recoveries obtained from the commercial human serum samples analysis.

Serum Sample	Added HbA1c (%)	Found HbA1c (%)	Recovery (%)
Sample 1	7.66	7.13 ± 0.09	93.09
Sample 2	5.50	5.41 ± 0.42	98.44
Sample 3	3.83	3.67 ± 0.35	95.72

**Table 3:** Comparison of the results obtained on various electrochemical aptasensors reported in the literature for HbA1c determination.

No.	Electrode (Platform)	Method	Limit of Detection	Linear Range	Incubation Time (with Target)	Sensitivity	Samples	Recovery (%)	Ref.
1.	SPE/AuNPs/APT	SWV	0.2 ng/mL	0.1–100 ng/mL	30 min	-	quality control samples of human whole blood	-	[8]
2.	SPE/pTBA@MWCNT/TBO/APT	Amperometric	3.7 nM	0.006–0.084 µM 0.084–0.74 µM	4 min	-	real blood samples	95	[9]
3.	GS/rGO-Au nanostructure/APT	DPV	1 nM	0.001–13.83 μM	60 min	269.2 μA/cm^2^	real blood samples	94.0–109.5	[10]
4.	SPE/APT	DPV	84 pg/mL	0.1–14 µg/mL	90 min	-	protein samples	-	[15]
5.	SPE/SWCNT/APT	SWV	0.03 pg/mL	0.1 pg/mL–1000 ng/mL	30 min	-	quality control samples of human whole blood	-	[16]
6.	CC/MIP/APT-Ag@HCS	DPV	0.35 μg/mL	0.8–78.4 μg/mL	-	4.365 μA/μg/mL/cm^2^	real human serum samples	98.7–99.6	[17]
7.	SPE/MoSe_2_/ Co-MOF/Anti-HbA1c/HbA1c/APT/SiW_12_	DPV	4.85 ng/mL	50–250 ng/mL	40 min	-	real human serum samples	98.89 –108.03	[18]
							commercial human serum samples	100–107.93	
8.	SPE/CD-CNTs/ AuNPs/APT	DPV	0.098%	0.688–11.5%	30 min	2.593 µA/%, 0.889 µA/%	commercial human serum samples	93.09–98.44	This work

SPE—screen-printed electrode; APT—aptamer; AuNPs—gold nanoparticles; pTBA—poly(2,2′:5′,5″-terthiophene-3′-p-benzoic acid); MWCNT—multi-walled carbon nanotube; TBO—toluidine blue O; GS—graphite sheet; rGO—reduced graphene oxide; SWCNT—single-walled carbon nanotube; CC—carbon cloth; MIP—molecularly imprinted polymer; Ag@HCS—Ag nanoparticle-doped hollow carbon nanospheres; Co-MOF—cobalt-based metal–organic framework; CD—cyclodextrin; DPV—differential pulse voltammetry; SWV—square wave voltammetry.

## Data Availability

Data are contained within the article.

## References

[1] Teymourian H., Barfidokht A., Wang J. (2020). Electrochemical Glucose Sensors in Diabetes Management: An Updated Review (2010–2020). Chem. Soc. Rev..

[2] Singh V., Krishnan S. (2018). Electrochemical and Surface Plasmon Insulin Assays on Clinical Samples. Analyst.

[3] Gupta S., Chauhan N., Jain U. (2017). Laboratory Diagnosis of HbA1c: A Review. J. Nanomed. Res..

[4] Lin H., Yi J. (2017). Current Status of HbA1c Biosensors. Sensors.

[5] Zhou Y., Dong H., Liu L., Hao Y., Chang Z., Xu M. (2015). Fabrication of Electrochemical Interface Based on Boronic Acid-Modified Pyrroloquinoline Quinine/Reduced Graphene Oxide Composites for Voltammetric Determination of Glycated Hemoglobin. Biosens. Bioelectron..

[6] Li J., Chang K.-W., Wang C.-H., Yang C.-H., Shiesh S.-C., Lee G.-B. (2016). On-Chip, Aptamer-Based Sandwich Assay for Detection of Glycated Hemoglobins via Magnetic Beads. Biosens. Bioelectron..

[7] Chang K.-W., Li J., Yang C.-H., Shiesh S.-C., Lee G.-B. (2015). An Integrated Microfluidic System for Measurement of Glycated Hemoglobin Levels by Using an Aptamer–Antibody Assay on Magnetic Beads. Biosens. Bioelectron..

[8] Eissa S., Zourob M. (2017). Aptamer-Based Label-Free Electrochemical Biosensor Array for the Detection of Total and Glycated Hemoglobin in Human Whole Blood. Sci. Rep..

[9] Moon J.-M., Kim D.-M., Kim M.H., Han J.-Y., Jung D.-K., Shim Y.-B. (2017). A Disposable Amperometric Dual-Sensor for the Detection of Hemoglobin and Glycated Hemoglobin in a Finger Prick Blood Sample. Biosens. Bioelectron..

[10] Shajaripour Jaberi S.Y., Ghaffarinejad A., Omidinia E. (2019). An Electrochemical Paper Based Nano-Genosensor Modified with Reduced Graphene Oxide-Gold Nanostructure for Determination of Glycated Hemoglobin in Blood. Anal. Chim. Acta.

[11] Zhang P., Zhang Y., Xiong X., Lu Y., Jia N. (2020). A Sensitive Electrochemiluminescence Immunoassay for Glycosylated Hemoglobin Based on Ru(Bpy)32+ Encapsulated Mesoporous Polydopamine Nanoparticles. Sens. Actuators B Chem..

[12] Almusharraf A.Y., Eissa S., Zourob M. (2018). Truncated Aptamers for Total and Glycated Hemoglobin, and Their Integration into a Graphene Oxide-Based Fluorometric Method for High-Throughput Screening for Diabetes. Microchim. Acta.

[13] Sun D., Wu Y., Chang S.-J., Chen C.-J., Liu J.-T. (2021). Investigation of the Recognition Interaction between Glycated Hemoglobin and Its Aptamer by Using Surface Plasmon Resonance. Talanta.

[14] Pohanka M. (2021). Glycated Hemoglobin and Methods for Its Point of Care Testing. Biosensors.

[15] Feng X.-Q., Ju Y., Dou W.-T., Li Q., Jin Z.-G., He X.-P., James T.D., Ye B.-C. (2021). Ferrocene-Labelled Electroactive Aptamer-Based Sensors (Aptasensors) for Glycated Haemoglobin. Molecules.

[16] Eissa S., Almusharraf A.Y., Zourob M. (2019). A Comparison of the Performance of Voltammetric Aptasensors for Glycated Haemoglobin on Different Carbon Nanomaterials-Modified Screen Printed Electrodes. Mater. Sci. Eng. C.

[17] Yang Y., Dong H., Yin H., Gu J., Zhang Y., Xu M., Wang X., Zhou Y. (2023). Controllable Preparation of Silver-Doped Hollow Carbon Spheres and Its Application as Electrochemical Probes for Determination of Glycated Hemoglobin. Bioelectrochemistry.

[18] Anuthum S., Papan P., Pasena A., Yimklan S., Aramrat C., Sangthong P., Jakmunee J., Ounnunkad K. (2025). Sensitive Electrochemical Detection of Glycated Hemoglobin (HbA1c) Using Cobalt Metal-Organic Framework/Two-Dimensional Molybdenum Diselenide Nanocomposite-Based Immunosensors Amplified by Polyoxometalate/DNA Aptamer. Colloids Surf. B Biointerfaces.

[19] Zhan Z., Li Y., Zhao Y., Zhang H., Wang Z., Fu B., Li W.J. (2022). A Review of Electrochemical Sensors for the Detection of Glycated Hemoglobin. Biosensors.

[20] Sharma P., Panchal A., Yadav N., Narang J. (2020). Analytical Techniques for the Detection of Glycated Haemoglobin Underlining the Sensors. Int. J. Biol. Macromol..

[21] Mandali P.K., Prabakaran A., Annadurai K., Krishnan U.M. (2023). Trends in Quantification of HbA1c Using Electrochemical and Point-of-Care Analyzers. Sensors.

[22] Ku T.-H., Zhang T., Luo H., Yen T., Chen P.-W., Han Y., Lo Y.-H. (2015). Nucleic Acid Aptamers: An Emerging Tool for Biotechnology and Biomedical Sensing. Sensors.

[23] Ștefan G., Hosu O., De Wael K., Lobo-Castañón M.J., Cristea C. (2021). Aptamers in Biomedicine: Selection Strategies and Recent Advances. Electrochim. Acta.

[24] Li B., Ellington A.D., Fox K.R., Brown T. (2012). Electrochemical Techniques as Powerful Readout Methods for Aptamer-based Biosensors. DNA Conjugates and Sensors.

[25] Shaban S.M., Kim D.-H. (2021). Recent Advances in Aptamer Sensors. Sensors.

[26] Saberian-Borujeni M., Johari-Ahar M., Hamzeiy H., Barar J., Omidi Y. (2017). Nanoscaled Aptasensors for Multi-Analyte Sensing. BioImpacts.

[27] Hernandez F.J., Ozalp V.C. (2012). Graphene and Other Nanomaterial-Based Electrochemical Aptasensors. Biosensors.

[28] Maduraiveeran G., Sasidharan M., Ganesan V. (2018). Electrochemical Sensor and Biosensor Platforms Based on Advanced Nanomaterials for Biological and Biomedical Applications. Biosens. Bioelectron..

[29] Yang Y., Yang X., Yang Y., Yuan Q. (2018). Aptamer-Functionalized Carbon Nanomaterials Electrochemical Sensors for Detecting Cancer Relevant Biomolecules. Carbon.

[30] Pirzada M., Altintas Z. (2019). Nanomaterials for Healthcare Biosensing Applications. Sensors.

[31] Maduraiveeran G., Jin W., Hussain C.M. (2020). Functional nanomaterial-derived electrochemical sensor and biosensor platforms for biomedical applications. Handbook of Nanomaterials in Analytical Chemistry.

[32] Fritea L., Banica F., Costea T., Moldovan L., Dobjanschi L., Muresan M., Cavalu S. (2021). Metal Nanoparticles and Carbon-Based Nanomaterials for Improved Performances of Electrochemical (Bio)Sensors with Biomedical Applications. Materials.

[33] Fritea L., Tertiş M., Cristea C., Săndulescu R. (2023). Exploring the Research Progress about the Applications of Cyclodextrins and Nanomaterials in Electroanalysis. Electroanalysis.

[34] Javanbakht S., Darvishi S., Dorchei F., Hosseini-Ghalehno M., Dehghani M., Pooresmaeil M., Suzuki Y., Ul Ain Q., Ruiz Rubio L., Shaabani A. (2023). Cyclodextrin Host–Guest Recognition in Glucose-Monitoring Sensors. ACS Omega.

[35] Mincu N.-B., Lazar V., Stan D., Mihailescu C.M., Iosub R., Mateescu A.L. (2020). Screen-Printed Electrodes (SPE) for In Vitro Diagnostic Purpose. Diagnostics.

[36] Haddad R., Cosnier S., Maaref A., Holzinger M. (2009). Non-Covalent Biofunctionalization of Single-Walled Carbon Nanotubes via Biotin Attachment by π-Stacking Interactions and Pyrrole Polymerization. Analyst.

[37] Gao Q., Guo Y., Zhang W., Qi H., Zhang C. (2011). An Amperometric Glucose Biosensor Based on Layer-by-Layer GOx-SWCNT Conjugate/Redox Polymer Multilayer on a Screen-Printed Carbon Electrode. Sens. Actuators B Chem..

[38] Adumitrăchioaie A., Tertiș M., Suciu M., Graur F., Cristea C. (2019). A Novel Immunosensing Platform for Serotonin Detection in Complex Real Samples Based on Graphene Oxide and Chitosan. Electrochim. Acta.

[39] Irimes M.-B., Tertis M., Bogdan D., Diculescu V., Matei E., Cristea C., Oprean R. (2024). Customized Flexible Platform—Starting Point for the Development of Wearable Sensor for the Direct Electrochemical Detection of Kynurenic Acid in Biological Samples. Talanta.

[40] Masood Z., Muhammad H., Tahiri I.A. (2024). Comparison of Different Electrochemical Methodologies for Electrode Reactions: A Case Study of Paracetamol. Electrochem.

[41] Fritea L., Bănică F., Costea T.O., Moldovan L., Iovan C., Cavalu S. (2018). A Gold Nanoparticles—Graphene Based Electrochemical Sensor for Sensitive Determination of Nitrazepam. J. Electroanal. Chem..

[42] Tertis M., Zăgrean M., Pusta A., Suciu M., Bogdan D., Cristea C. (2023). Innovative Nanostructured Aptasensor for the Electrochemical Detection of Gluten in Food Samples. Microchem. J..

[43] Malecka-Baturo K., Zaganiaris A., Grabowska I., Kurzątkowska-Adaszyńska K. (2022). Electrochemical Biosensor Designed to Distinguish Tetracyclines Derivatives by ssDNA Aptamer Labelled with Ferrocene. Int. J. Mol. Sci..

[44] Kim G., Park S.E., Lee W., Joo J.M., Yang H. (2024). Ferrocenyl Compounds as Alternative Redox Labels for Robust and Versatile Electrochemical Aptamer-Based Sensors. ACS Sens..

[45] Toh R.J., Peng W.K., Han J., Pumera M. (2014). Direct In Vivo Electrochemical Detection of Haemoglobin in Red Blood Cells. Sci. Rep..

[46] Yuan Y., Ni X., Cao Y. (2019). Electrochemical Determination of Hemoglobin on a Magnetic Electrode Modified with Chitosan Based on Electrocatalysis of Oxygen. J. Electroanal. Chem..

[47] Zhu L., Li X., Deng Y., Zou R., Shao B., Yan L., Ruan C., Sun W. (2021). Construction and Electrochemical Behavior of Hemoglobin Sensor Based on ZnO Doped Carbon Nanofiber Modified Electrode. J. Iran. Chem. Soc..

[48] Thiruppathi M., Lee J.-F., Chen C.C., Ho J.A. (2021). A Disposable Electrochemical Sensor Designed to Estimate Glycated Hemoglobin (HbA1c) Level in Whole Blood. Sens. Actuators B Chem..

[49] Ponsanti K., Ngernyuang N., Tangnorawich B., Na-Bangchang K., Boonprasert K., Tasanarong A., Saeheng T., Hanwattanakul A., Pechyen C. (2022). A Novel Electrochemical-Biosensor Microchip Based on MWCNTs/AuNPs for Detection of Glycated Hemoglobin (HbA1c) in Diabetes Patients. J. Electrochem. Soc..

[50] Shen Q., Wang X. (2009). Simultaneous Determination of Adenine, Guanine and Thymine Based on β-Cyclodextrin/MWNTs Modified Electrode. J. Electroanal. Chem..

[51] Gilbert S., Jeong H.K., Dowben P.A. (2017). Cyclodextrin-Carbon Nanotube Composites for Fluorescent Detection of Cholesterol. Chem. Phys. Lett..

[52] Le H.T.N., Jeong H.K. (2017). Cyclodextrin-Graphite Oxide-Carbon Nanotube Composites for Electrochemical Supramolecular Recognition. Electrochim. Acta.

[53] Lin H.-I., Wu C.-C., Yang C.-H., Chang K.-W., Lee G.-B., Shiesh S.-C. (2015). Selection of Aptamers Specific for Glycated Hemoglobin and Total Hemoglobin Using On-Chip SELEX. Lab Chip.

